# From conceptual pluralism to practical agreement on policy: global responsibility for global health

**DOI:** 10.1186/s12914-015-0065-8

**Published:** 2015-10-28

**Authors:** Jennifer Prah Ruger, Rachel Hammonds, Gorik Ooms, Donna Barry, Audrey Chapman, Wim Van Damme

**Affiliations:** Perelman School of Medicine and Leonard Davis Institute of Health Economics, University of Pennsylvania, Philadelphia, USA; Institute of Tropical Medicine, Law and Development Research Group, University of Antwerp, Antwerp, Belgium; Center for American Progress, Washington, DC USA; University of Connecticut Health Center, Farmington, USA

**Keywords:** Global Fund to Fights AIDS Tuberculosis and Malaria, Global Fund for Health, Development Assistance for Health, Global Health

## Abstract

**Background:**

As the human cost of the global economic crisis becomes apparent the ongoing discussions surrounding the post-2015 global development framework continue at a frenzied pace. Given the scale and scope of increased globalization moving forward in a post-Millennium Development Goals era, to protect and realize health equity for all people, has never been more challenging or more important. The unprecedented nature of global interdependence underscores the importance of proposing policy solutions that advance realizing global responsibility for global health.

**Discussion:**

This article argues for advancing global responsibility for global health through the creation of a Global Fund for Health. It suggests harnessing the power of the exceptional response to the combined epidemics of AIDS, TB and Malaria, embodied in the Global Fund to Fight AIDS, Tuberculosis and Malaria, to realize an expanded, reconceptualized Global Fund for Health. However this proposal creates both an analytical quandary embedded in conceptual pluralism and a practical dilemma for the scope and raison d’etre of a new Global Fund for Health. To address these issues we offer a logical framework for moving from conceptual pluralism in the theories supporting global responsibility for health to practical agreement on policy to realize this end. We examine how the innovations flowing from this exceptional response can be coupled with recent ideas and concepts, for example a global social protection floor, a Global Health Constitution or a Framework Convention for Global Health, that share the global responsibility logic that underpins a Global Fund for Health.

**Conclusions:**

The 2014 Lancet Commission on Global Governance for Health Report asks whether a single global health protection fund would be better for global health than the current patchwork of global and national social transfers. We concur with this suggestion and argue that there is much room for practical agreement on a Global Fund for Health that moves from the conceptual level into policies and practice that advance global health. The issues of shared responsibility and mutual accountability feature widely in the post-2015 discussions and need to be addressed in a coherent manner. Our article argues why and how a Global Fund for Health effectuates this, thus advancing global responsibility for global health.

## Background

The impact of the global economic crisis on the health of the most vulnerable people of the world has become starkly apparent [1], and a matter of increasingly urgent concern. Given the scale and scope of the current situation and the process of moving forward in a post-Millennium Development Goals era, protecting and realizing health for all people has never been more challenging or more important. Global initiatives are ongoing that are taking on this challenge [2]. The crisis and current situation illustrates an unprecedented global interdependence and underscores the global responsibility for global health.

Until now, global responsibility in global health and practical policy responses such as the Global Fund to Fight AIDS, Tuberculosis and Malaria (GFATM) model have been driven in great part by the perceived exceptionality of the combined epidemic of AIDS, TB and Malaria, and an accepted global responsibility to turn the tide of this epidemic. This exceptionality is now being questioned, and as a result, so is the global responsibility. This elucidates why some donor countries proposed caps on future grant rounds; there is a growing reluctance of rich countries to mobilize resources.

This article argues that there are alternatives to the exceptionality of the combined epidemic of AIDS, TB and Malaria, alternatives that also support the idea of global responsibility for health, and a Global Fund For Health expressing this global responsibility. However, these alternatives typically ground global responsibility for health realized practically through a Global Fund for Health in only single theoretical concepts: “political realism” [3] or “the right to health” [1], to highlight a few and do not support a Global Fund for Health with a mandate limited to three diseases. This creates both an analytical quandary embedded in conceptual pluralism and a practical dilemma for the scope and raison d’etre of the GFATM. Analytically, at first glance it is difficult to see how to construct practical policies out of seemingly intractable conceptual variety. In this article, therefore, we provide something that is missing in the theoretical space in global health: a logical framework that allows moving from conceptual pluralism in theories supporting global responsibility for health to practical agreement on policy to realize this end. Practically, the problem for the GFATM is that its mandate and rasion d’etre are in question and it might not have the resources needed to embrace a wider set of responsibilities. UNAIDS Executive Director Michel Sidibé echoes this concern stating that “the international community cannot afford to sustain the current architecture for AIDS and global health in the coming years” [4]. This article argues that if the GFATM continues to ground its ‘raison d’etre’ on the exceptionality of the combined epidemic of AIDS, TB and Malaria, it risks going down together with the fall of this exceptionality. To respond to these challenges we propose a solution: embrace a wider mandate, and a reformulated raison d’etre, one that is grounded in broader notions of global responsibility for global health, and become a Global Fund for Health. While it is not within the scope of this article to suggest the practical means necessary for realigning the preferences and commitments of the various stakeholders in global health governance to establish a Global Fund for Health, we demonstrate that it is in principle possible to move from conceptual pluralism in this matter to practical agreement on policy.

## Discussion

### Global responsibility and global health

In recent years, ideas and concepts of global responsibility for global health have been put forward, resulting in conceptual pluralism around this theme: a ‘world social health insurance’ [5, 6]; ‘globalisation of social protection’ [7–9] or a ‘global social protection floor’ [10]; ‘health security’, understood as the health components of ‘human security’ [11, 12]; a ‘provincial globalism’ theory of ‘global health justice’ [13, 14], encompassing universal coverage of comprehensive efforts to improve health and integrating aspects of human rights, protection and security [15]; a ‘framework convention on global health’ [16]; a new ‘Monterrey consensus’ to engage emerging powers in shared responsibility for development [17]; ‘shared health governance’ and a “global health constitution’ [18, 19], to mention a few. This article argues for moving these concepts into policy and practice.

### Moving from conceptual pluralism to practical agreement on policy

Whether one starts from the concept of human rights, global social protection, or human security, when moving from concepts to practice, it is possible to obtain practical agreement on policy options, without attenuating the fundamental differences between these concepts. To make this case, we adopted Jennifer Prah Ruger’s idea of ‘incomplete theorisation in health’. It may very well be possible, Ruger argues, to completely theorize a particular policy, program, proposal, or intervention all the way from high level values to low level particulars. But more often than not, agreement is possible at one or more levels and on one or more dimensions, without agreement on other levels or dimensions [20].

**Fig. 1:**
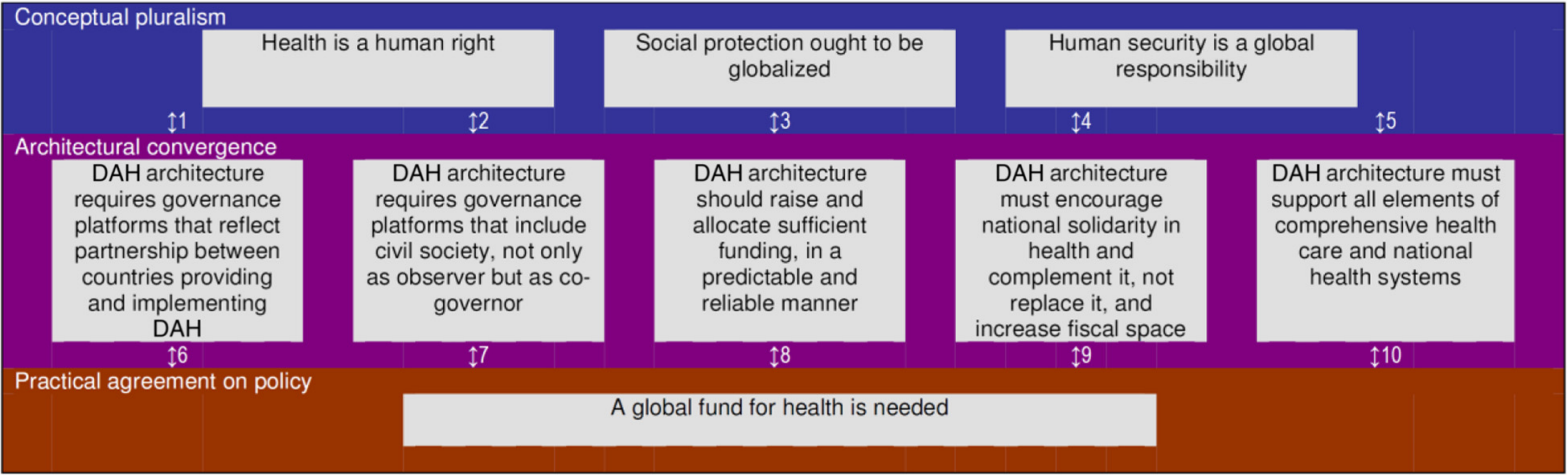
Global responsibilities for global health, from concepts to practice

In recent years various global health initiatives including horizontal initiatives (focusing on health systems), vertical interventions (targeting specific diseases like HIV/AIDS) and vertical-horizontal combinations have attempted to improve global health outcomes. But the multiplicity of actors on the global health stage has resulted in inefficiencies which fail to address and even perpetuate inequity in health outcomes. Acknowledging the impact of poor coordination in improving outcomes, the International Health Partnership and related initiatives (IHP+) seeks to achieve better health results by harmonizing bilateral donors and other development partners around a single country-led national health ‘compact’ [21]. We support this move towards harmonization of Development Assistance for Health (DAH), but argue that there is a need to go further and to address the architecture itself, practical agreement on the need for a Global Fund for Health, as one of the best policy options for global health, is possible. Figure 1 illustrates this. We will briefly explain each of the 10 linkages in this figure. Linkages one through five demonstrate how each of the concepts supporting global responsibility for health leads to essential features of a better DAH architecture. Linkages six to ten show how these essential features lead to the creation of a Global Fund for Health, as a cornerstone of the DAH architecture.

For the first linkage, whether one starts from the concept of the right to health, global social protection, or human security, the DAH architecture should provide equal power in governance, between countries contributing DAH and those implementing it, replacing the present discretionary power of countries contributing DAH to decide what they want to support and what they do not want to support. Dybul and Frenk’s discussion on the importance of reflecting the new global economic and political reality argues for productive engagement with emerging powers to redress past errors and move towards shared responsibility and mutual accountability [17]. Related to this feature is the importance of country-specific policies, meaning an architecture that is sufficiently flexible to avoid ‘one size fits all’ solutions and to allow for countries to think ambitiously in terms of funding.

In the second linkage, civil society participation from the inception of DAH-supported health care to the monitoring of its implementation is important, whichever concept one employs. Under a right to health approach, countries implementing DAH do so on behalf of their inhabitants, who are the ultimate rights-holders. In a social protection scheme, beneficiaries are also participants. The human security approach aims to ensure the basic security of all humans. These approaches are fundamentally different from approaches based on humanitarianism, generosity or charity from one state to another.

For the third linkage, the DAH architecture should address problems in the volume of DAH. (Only a few countries allocate the equivalent of 0.7 % of Gross Domestic Product to international assistance, despite repeated affirmations of this commitment). The long-term predictability of funding needs to be addressed. Failure on these points has meant that countries implementing DAH reluctant to plan long term for fear that the next funding cycle will result in shortfalls.

In the fourth linkage, there is a need to encourage expanding domestic budgetary allocations in countries implementing DAH. Expanding so-called ‘fiscal space’ requires both long-term DAH predictability and increasing domestic revenue. It requires moving beyond old policies and agendas that defined fiscal space too narrowly. Growing domestic economies (and ensuring that national treasuries benefit through increased tax revenue) is vital if the Abuja Declaration’s pledge – 15 % of the budget for the health sector [22] – is to be met and if met it is not to be 15 % of a very small amount.

On the fifth linkage, the DAH architecture must promote all elements of comprehensive efforts to improve health, including national health systems (public health and health care) capable of providing these efforts in an equitable manner, without pitting one health problem against another.

The sixth linkage relates to the Board of the GFATM, in which countries contributing DAH and countries implementing DAH are equally represented, and which provides an example of partnership that is needed for advancing global health. Transforming the current GFATM into a Global Fund for Health would be a practical way to achieve this by resulting in board representation by all countries, not just those fighting AIDS, tuberculosis, and malaria, thus reducing the inequities and power disparities associated with the current DAH regime.

The seventh linkage involves preserving and reinforcing the so-called ‘Country Coordination Mechanisms’ (CCMs) in the new Global Fund for Health, which would also allow giving civil society a voice in the DAH architecture. While CCMs are not free of problems, they are useful platforms for civil society participation, which is necessary if we are to benefit from the voices and perspectives of those whose health is ostensibly to be improved through initiatives like a Global Fund for Health [23]. The oversight bodies for ‘Sector-Wide Approach’ (SWAPs), or related ‘basket funds’, do not usually foresee such mechanisms, but the basic notion could be revised going forward to include the participation of civil society.

For the eighth linkage, using estimates of the High Level Taskforce on Innovative International Financing for Health Systems (Taskforce), the DAH architecture would need to raise and allocate US$50 billion annually [21]. Although the Taskforce did not use any of the above mentioned concepts supporting a global responsibility for health, it is unlikely that the use of any of these concepts would lead to a lower estimate. This level of DAH should be predictable and reliable, and thus it would be preferable that contributions be required, or at least be subjected to peer pressure (for example, contributions to the International Development Association) and mutual accountability. Achieving this level of DAH would require about 0.1 % of the combined GDP of high-income countries, or 15 % of the prior commitment to allocate 0.7 % of GDP to international aid (mirroring the commitment made in the Abuja Declaration). Some fluctuations in DAH contributions will be inevitable, if only because the GDP of contributing countries fluctuates. To create a buffer for fluctuations and to spread unexpected shortfalls evenly over countries implementing DAH, it seems advisable to pool all or the majority of DAH in a Global Fund for Health.

The ninth linkage underscores that DAH should not replace national health responsibility – the domestic commitment to health justice [13, 15] – DAH should encourage and support national health solidarity. Global responsibility would not replace national responsibility. Reasonable levels of national health morality in countries implementing DAH must therefore be agreed upon, and these countries would have to be accountable for their progress in this respect, as much as countries providing DAH would be held accountable for meeting their commitments (see linkage 8). This should be understood as an element of the partnership and a provincial-global consensus on health morality [13]. The resulting mutual commitment and the budget allocations supporting it should convince international financial institutions that there is sufficient fiscal space for substantially increased government health expenditure. One must be careful to avoid the reduced efforts in expanding domestic budgetary allocations, as the result of not wanting to scale up public expenditure, while not forfeiting the available DAH. Pooling funds in a Global Fund for Health should increase predictability (see linkage 8) and increase the likelihood that DAH is truly additional to domestic expenditure thus satisfying the objectives of all partners [3].

Last but not least, for the tenth linkage the Global Fund for Health would provide DAH for comprehensive health efforts, not only for efforts against specific health conditions like AIDS, tuberculosis, and malaria which may not result in strengthened public health systems or improved general well-being for all.

### Objections and future options

Some will argue that a Global Fund for Health may be desirable, but not essential. Could the International Health Partnerships (plus Related Initiatives) and the ‘compacts’ it aims for not provide all the features mentioned above? We argue that it does not. Let us use the example of the health compact, elaborated by the Ministry of Health of Ethiopia, within the context of the IHP+. The cost is estimated at US$1.9 billion per year (for 80 million people, still less than US$25 per person per year). This estimate has been validated by the World Health Organization (WHO), the World Bank, United Nations FPA, UNICEF and several donor countries in the form of a ‘Joint Financial Arrangement’, according to which Ethiopia would need to spend US$1.4 billion per year on health care in addition to the US$0.5 billion it spends on health care at present [24]. An effort like this would have to be sustained over many years, if not decades, as it would not make sense to increase the budget from US$0.5 billion to US$1.9 billion and return to US$0.5 billion after a few years. It seems unlikely that a US$1.4 billion DAH budget will come from a patchwork of bilateral agreements in a sufficiently reliable manner; even if it did so, it would be an administrative quagmire.

In a practical example, if a Global Fund for Health existed, then the compact would have been submitted to a CCM (with a wider mandate and a larger composition), where civil society would be able to participate in amending it. It would then go to an independent technical review panel, which would evaluate the compact on its technical merits and verify if all elements of comprehensive primary health care are included. If approved, the Board of the Global Fund for Health – where countries providing DAH and countries implementing DAH would be equally represented – would allow the secretariat to transform the compact into a financial agreement, including the DAH contribution and the domestic contribution. The Global Fund for Health would need much more resources than the present GFATM has; so an agreement on burden-sharing between countries providing DAH would be a prerequisite.

There are, in broad terms, two methods for designing a DAH architecture that would meet these conditions. One is to design something new, as noted above. The other is to assess the current architecture and propose incremental changes, towards an improved design.

Examining the current DAH architecture to find potential incremental changes, one can distinguish three different types of DAH channels: global health initiatives, bilateral DAH channels, and World Bank funding. Global health initiatives should adapt their governance platforms in line with the requirements mentioned above. They should seriously consider expanded mandates and mergers to consolidate the quantity. The existence of more than a hundred global health initiatives, or even just twenty-three, depending on the definition one employs [25], makes the architecture unnecessarily complex. Through a series of mergers, these initiatives could themselves become a Global Fund for Health. Bilateral DAH channels should question whether it is at all possible to move towards a relationship of equal partnership in bilateral assistance and to include civil society in a meaningful way. The flexibility bilateral DAH channels provide will remain an essential feature of any DAH architecture but for core DAH, such as the Ethiopia compact, they should consider pooling more DAH into a Global Fund for Health. The World Bank should examine the kind of governance that currently steers the DAH it provides – a Board of Governors, with votes in accordance with countries’ economic power – and consider the creation of a specific governance body to oversee its DAH. DAH should conform to principles of global health justice. To improve prospects for global health equity, DAH should abandon the donor-recipient dichotomy and replace it with equal respect for all person’s capabilities [26].

Sidibé has argued that the “the Global Fund is well-positioned to evolve into a more stream-lined mechanism for global health financing beyond AIDS, tuberculosis and malaria that could deliver greater coherence and equity across the post-2015 landscape” [4]. Some have expressed fears about a rapid expansion of the GFATM’s mandate without prior guarantees about additional funding. Notably, one critic has written: “Without the necessary additional funding, this proposition will just water down the Global Fund’s current ability to deliver effectively and make an impact” [27]. However, supporters of a Global Fund for Health point out that success in addressing priority diseases is “intrinsically fragile” in the absence of strong health systems [28]. Both specific conditions and health systems must be addressed to achieve long-term positive change. We agree, however, that a GFATM with a wider mandate would need a lot more funding to remain functional, but one might remember how the creation of the GFATM thirteen years ago required a ‘leap of faith’, overcoming fears then that it would mainly shift existing DAH, not add to it [29]. So-called ‘AIDS exceptionalism’ was useful in spurring an alternative way of thinking about global health, but none of the theoretical concepts undergirding global health can justify prioritizing one disease above all others [30]. Global health may require an additional ‘leap of faith’.

## Conclusions

This article argues for advancing global responsibility for global health through a Global Fund for Health which builds on the exceptional response to the combined epidemic of AIDS, TB and Malaria, embodied by the GFTAM. We argue that if the GFATM continues to ground its ‘raison d’etre’ on exceptionality related to three diseases, it risks going down together with the fall of this exceptionality. However expanding the mandate of the GFTAM to realize an expanded, reconceptualized Global Fund for Health creates both an analytical quandary embedded in conceptual pluralism and a practical dilemma. In addressing these issues we offer a logical framework for moving from conceptual pluralism to practical agreement on policy. We examine how the innovations flowing from this exceptional response can be coupled with recent innovative ideas and concepts addressing global responsibility for global health. These recent propositions have an overlapping focus on shared responsibility and mutual accountability suggesting there is much room for practical agreement and the possibility of moving from the conceptual level into policies and practice that advance global health. We argue that supporting a Global Fund for Health would be a strong expression of support for global responsibility for global health.

## References

[1] Ooms G, Hammonds R (2008). Correcting globalisation in health: transnational entitlements versus the ethical imperative of reducing aid-dependency. Public Health Ethics.

[2] Ottersen OP, Frenk J, Horton R (2011). The Lancet—University of Oslo Commission on Global Governance for Health, in collaboration with the Harvard Global Health Institute. Lancet.

[3] Ooms G, Hammonds R (2014). Financing global health through a Global Fund for Health? Chatham House Working Paper.

[4] Sidibe M. Time to demand a new financing paradigm for AIDS, global health and development. Global Health and Diplomacy; Summer 2014:13–15.

[5] Van Damme W (2007). World social health insurance: strengthening health systems in low-income countries. PLoS Med.

[6] Ooms G, Derderian K, Melody D (2006). Do we need a world health insurance to realise the right to health?. PLoS Med.

[7] Van Langendonk J, Van Langendonck J (2007). The meaning of the right to social security. The right to social security.

[8] Ottersen OP, Dasgupta J, Blouin C, Buss P, Chongsuvivatwong V, Frenk J (2014). The political origins of health inequality, prospects for change. Lancet.

[9] De Schutter O, Sepúlveda M (2012). Underwriting the poor—a global fund for social protection.

[10] ILO (2011). Social protection for a fair and inclusive globalization. Report of the Social Protection Floor Advisory Group.

[11] Frenk J (2009). Strengthening health systems to promote security. Lancet.

[12] Takemi K, Jimba M, Ishii S, Katsuma Y, Nakamura Y (2008). Human security approach for global health. Lancet.

[13] Ruger JP (2009). Global health justice. Public Health Ethics.

[14] Ruger JP (2006). Ethics and governance of global health inequalities. J Epidemiol Community Health.

[15] Ruger JP (2009). Health and social justice.

[16] Gostin L (2007). A proposal for a framework convention on global health. J Int Econ Law.

[17] Dybul M, Frenk J. A new development framework. The Global Fund to Fight AIDS, Tuberculosis and Malaria, Jan 9 2014. Available from: http://www.theglobalfund.org/en/blog/2014-01-09_A_New_Development_Framework/.

[18] Ruger JP (2012). Global health governance as shared health governance. J Epidemiol Community Health.

[19] Ruger JP (2013). A global health constitution for global health. Proceedings of the Annual Meeting. Am Soc Int Law.

[20] Ruger JP, Ruger JP, Ruger JP (1998). Aristotelian justice and health policy: capability and incompletely theorized agreements. Ph.D. Dissertation.

[21] Taskforce on Innovative International Financing for Health Systems (2009). More money for health and more health for the money.

[22] Organisation of African Unity (2001). Abuja declaration on HIV/AIDS, tuberculosis and other related infectious diseases.

[23] Garmaise D, Wafula A, Reisdorf K, Kageni A. Conflict of interest in country co-ordinating mechanisms. Aidspan 2013. Available from: http://aidspan.org.

[24] Ethiopian Ministry of Health Ethiopia. Joint statement of the Government of Ethiopia and Signatory Development Partners. 2009. Available from: http://www.internationalhealthpartnership.net/pdf/IHP%20Update%2013/Zambia/Joint%20Statement%20of%20The%20Government%20Of%20Ethiopia%20and%20Signatory%20Development%20Partners.pdf.

[25] Buse K, Harmer A (2007). Seven habits of highly effective global public-private health partnerships: practice and potential. Soc Sci Med.

[26] Ruger JP (2015). Ethics of development assistance for health. Hastings Cent Rep.

[27] Bermejo A (2009). Towards a global fund for the health MDGs?. Lancet.

[28] Cometto G, Ooms G, Starrs A, Zeitz P (2009). Towards a global fund for the health MDGs?. Lancet.

[29] Brugha R, Walt G (2001). A global health fund: a leap of faith?. BMJ.

[30] Ooms G (2008). Shifting paradigms: how the fight for ‘universal access to AIDS treatment and prevention’ supports achieving ‘comprehensive primary health care for all’. Global Health.

